# Axonal Regeneration by Glycosaminoglycan

**DOI:** 10.3389/fcell.2021.702179

**Published:** 2021-06-16

**Authors:** Kazuma Sakamoto, Tomoya Ozaki, Kenji Kadomatsu

**Affiliations:** ^1^Department of Biochemistry, Nagoya University Graduate School of Medicine, Nagoya, Japan; ^2^Institute for Glyco-Core Research (iGCORE), Nagoya University, Nagoya, Japan

**Keywords:** chondroitin sulfate, heparan sulfate, axonal regeneration, PTPσ, autophagy, dystrophic endbulb

## Abstract

Like other biomolecules including nucleic acid and protein, glycan plays pivotal roles in various cellular processes. For instance, it modulates protein folding and stability, organizes extracellular matrix and tissue elasticity, and regulates membrane trafficking. In addition, cell-surface glycans are often utilized as entry receptors for viruses, including SARS-CoV-2. Nevertheless, its roles as ligands to specific surface receptors have not been well understood with a few exceptions such as selectins and siglecs. Recent reports have demonstrated that chondroitin sulfate and heparan sulfate, both of which are glycosaminoglycans, work as physiological ligands on their shared receptor, protein tyrosine phosphatase sigma (PTPσ). These two glycans differentially determine the fates of neuronal axons after injury in our central nervous system. That is, heparan sulfate promotes axonal regeneration while chondroitin sulfate inhibits it, inducing dystrophic endbulbs at the axon tips. In our recent study, we demonstrated that the chondroitin sulfate (CS)-PTPσ axis disrupted autophagy flux at the axon tips by dephosphorylating cortactin. In this minireview, we introduce how glycans work as physiological ligands and regulate their intracellular signaling, especially focusing on chondroitin sulfate.

## Introduction

The human neural circuit, composed of the central nervous system (CNS) and the peripheral nervous system (PNS), reaches approximately 1 million km and is formed mainly by neural axons. An axon is a structure that is elongated from the cell body and relays information to its target cells, including neurons, glands, and muscles, by conducting electrical pulses and releasing neurotransmitters.

Axonal injury to our mature CNS, including spinal cord injury and traumatic brain injury, is still an untreatable condition, even with current medical knowledge. It was already recognized and described as “incurable” in the Edwin Smith Surgical Papyrus published between 2,500 and 1,900 BC in Egypt (Hughes, 1988). Emerging evidence has revealed that the lack of trophic factors and the existence of inhibitory environmental cues in the adult CNS made it difficult for nerve axons to spontaneously regenerate. Once damaged, axons enter a dormant state. Regeneration is possible only under certain circumstances.

Upon injury, the distal parts of axons that are separated from the cell body, undergo Wallerian degeneration. The fragmented and degenerated axonal shafts are phagocytosed and removed by microglia or macrophages. The process seems to be important for the regeneration of axons by the surviving neurons. In PNS, Wallerian degeneration is mediated mainly by peripheral macrophages and is accomplished quickly and completely, which is thought to be a factor in the high regeneration capacity of PNS axons. In the CNS, on the other hand, Wallerian degeneration is largely delayed and finished incompletely, probably because of the poor phagocytic capacity of microglia, major phagocytic cells in the CNS. In the 1980s, Aguayo and his colleagues clearly demonstrated that CNS axons regenerated through the implanted sciatic nerve “bridge,” confirming that the difference in environmental cues between the CNS and the PNS defined each axon’s regeneration capability (David and Aguayo, 1981; Benfey and Aguayo, 1982).

In addition to Wallerian degeneration, matrix remodeling after injury largely differs between the CNS and the PNS. In the CNS, the lesion site is surrounded by activated astrocytes (reactive astrocytes), forming a so-called glial scar (Silver and Miller, 2004). This scar is important for fixing the damaged blood-brain barrier (BBB), covering the lesion, protecting tissues from infections, and secreting various regeneration factors (Anderson et al., 2016). However, the glial scar also produces chondroitin sulfate (CS) proteoglycans, which are molecules that inhibit axonal regeneration (Snow et al., 1990, 1991; Rauch et al., 1991; Asher et al., 2000). When the regenerating axon tip makes contact with the CS proteoglycan (CSPG), it stops further extension and enters a dormant state called the dystrophic endbulb.

In this review, we briefly summarize how glycans work as physiological ligands and regulate axonal regeneration by mediating intracellular signaling.

## Dystrophic Endbulb

In 1928, Santiago Ramon Cajal found swollen axonal tips with multiple vacuoles in a lesion of a canine spinal cord. He reported that the structure was closely associated with poor axonal regeneration ability in the CNS. That structure is now recognized as the dystrophic endbulb or the dystrophic endball. The dystrophic endbulb contains a disorganized cytoskeleton and accumulations of membrane. It is formed acutely after injury can persist for several decades at lesions in human patients (Ruschel et al., 2015). Therefore, the structure was regarded as a therapeutic target for traumatic CNS injury, as Cajal suggested. However, the cellular and molecular mechanisms underlying the formation of dystrophic endbulb were unclear. Silver and his colleagues demonstrated that the increasing concentration gradient of CS that mimicked the *in vitro* glial scar was sufficient to induce dystrophic endbulbs on cultured adult dorsal root ganglion neurons (Tom et al., 2004). Defects in lysosomal secretion, including that of Cathepsin B, which might be important for matrix degradation and axonal elongation, were also suggested to be a characteristic of dystrophic endbulb (Tran et al., 2018; Tran and Silver, 2021).

## Roles of Glycosaminoglycans in CNS

**FIGURE 1:**
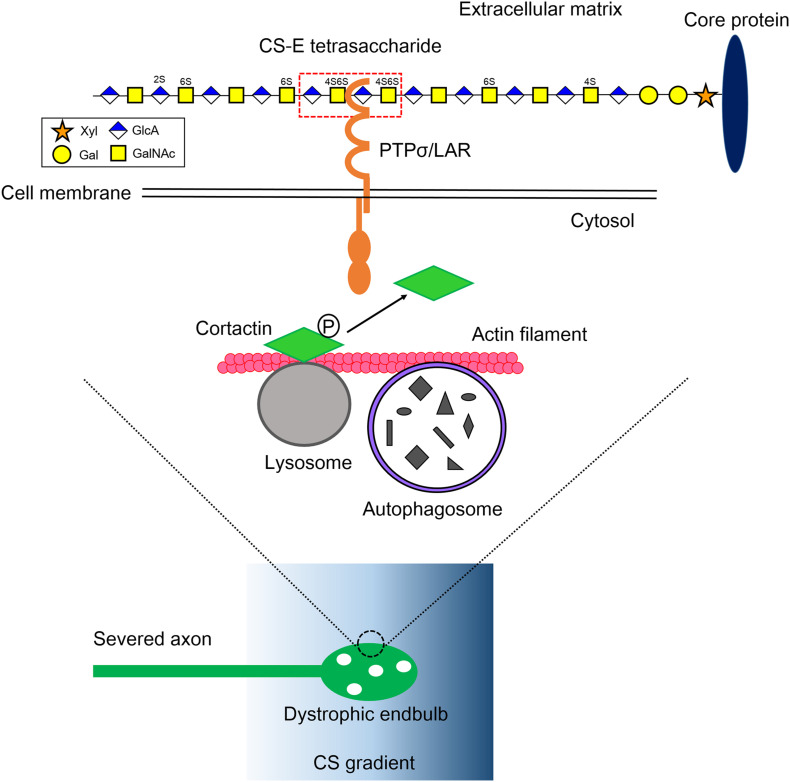
Mechanistic insight into the formation of dystrophic endbulb by CS. The severed axons run into the gradient of CS originated from the perineuronal net and the reactive astrocyte in the lesion. The CS-E tetrasaccharide segment, which rarely appears on a CS chain, preferentially monomerizes and activates its axonal receptor PTPσ/LAR. Contrary, HS protects the formation of dystrophic endball by inducing clustering of the receptors. RPTP dephosphorylates cortactin, which is localized onto the lysosome surface and stabilizes actin fibers to facilitate autolysosome formation. As a consequence, the CS-RPTP axis disrupts autophagy flux, inducing the failure of fusion between autophagosomes and lysosomes, and thus abnormal accumulation of autophagosomes. That leadsto transforms of axon tips to dystrophic endbulb. Xyl, xylose; Gal, galactose; GlcA, glucuronic acid; GalNAc, *N-*acetylgalactosamine; P, tyrosine phosphorylation.

CS is a glycosaminoglycan (GAG) and an unbranched polymer chain, which consists of the repeating disaccharide unit, glucuronic acid–*N*-acetylgalactosamine (GlcA-GalNAc) (Figure 1; Margolis and Margolis, 1993; Kadomatsu and Sakamoto, 2014; Sakamoto and Kadomatsu, 2017). In addition to CS, GAG contains heparan sulfate (HS), keratan sulfate, and hyaluronan. Except for HA, GAG is modified with sulfate groups and covalently attached to specific core proteins, forming proteoglycan (PG). HS is a linear polysaccharide and consists of repeating disaccharide unit, uronic acid and *N*-acetylglucosamine. The sequential modifications, *N*-deacetylation of *N*-acetylglucosamines, *N*-sulfation of glucosamines, and *O*-sulfations at the C2-position of uronic acids as well as C3- and/or C6-position of glucosamines can be occurred. Regarding its roles in axonal elongation and its inhibition, HS, which is a linear polysaccharide of repeated disaccharide of uronic acid and was revealed to be a positive regulator (Wang and Denburg, 1992; Aricescu et al., 2002). For example, mice lacking *EXT1*, one of the essential enzymes for the synthesis of HS, showed abnormal commissure formation of the corpus callosum (Inatani et al., 2003). On the other hand, CS and keratan sulfate were revealed to be negative regulators in axonal elongation. In addition to dystrophic endbulb-forming activity *in vitro* as described above, several works based on chondroitinase ABC, a CS-degrading enzyme with bacterial origin, clearly demonstrated that CS was involved in the inhibition of axonal regeneration after injury *in vivo.* Enzymatic digestion of CS side chains on PG by the enzyme dramatically enhanced both anatomical and functional plasticity after various SCI models (Moon et al., 2001; Bradbury et al., 2002). Importantly, combined with intermittent hypoxia, chondroitinase ABC promoted robust restoration of ventilation after SCI, the impairment of which is a major cause of mortality in human patients (Warren et al., 2018). A recent work also showed that C-ABC improved both anatomical and functional outcomes after spinal cord hemisection in monkey (Rosenzweig et al., 2019). Keratan sulfate was also demonstrated to inhibit axonal regeneration both *in vitro* and *in vivo* (Snow et al., 1990; Smith-Thomas et al., 1994; Ito et al., 2010; Imagama et al., 2011). It is noteworthy that CS and keratan sulfate often share proteoglycans, such as aggrecan and phosphacan (Rauch et al., 1991; Margolis and Margolis, 1993).

CS is a long glycan chain with approximately 50–100 saccharides on average. The C2-position of GlcA and the C4- and/or C6-positions of GalNAc can be sulfated, resulting in different disaccharide compositions including A-unit (GlcA-GalNAc4S), C-unit (GlcA-GalNAc6S), D-unit (GlcA2S-GalNAc6S), and E-unit (GlcA-GalNAc4S,6S), where 2S, 4S, and 6S stand for 2- O-, 4- O-, and 6-O-sulfate, respectively. Thus, a single chain of CS is heterogeneous in both length and structure and has been proposed to contain a “functional domain” to interact with other specific molecules (Mikami et al., 2009; Dickendesher et al., 2012). In mammalian CNS, CSPGs, neurocan, versican, brevican, aggrecan, NG2, and phosphacan, contribute to assemble into an extracellular matrix. Along with the development of the CNS, CS is enriched especially around inhibitory neurons and synapses in a well-organized manner, in what is known as a “perineurnal net,” where it regulates synaptic plasticity (Pizzorusso et al., 2002, 2006; Frischknecht et al., 2009; Gogolla et al., 2009; Miyata et al., 2012). Upon injury, this organized CS matrix might be disrupted and diffused in a disorganized manner and, together with CS newly synthesized by the glial scar, inhibits regeneration of the dissected axons, transforming the dystrophic endbulb at its tip (Silver and Miller, 2004). However, the action mechanisms of CS on axons remained unclear for about two decades after CS was recognized as a major inhibitory cue for axonal regeneration in our CNS. This was largely because specific neuronal receptors for CS had not been identified.

## Protein Tyrosine Phosphatase Sigma Functions as a CS Receptor

It was a big surprise that protein tyrosine phosphatase sigma (PTPσ) and leukocyte common antigen-related (LAR) were identified as neuronal receptors for CS (Shen et al., 2009; Fisher et al., 2011), because these two molecules had already been reported as receptors for HS and found to be positively involved in axon guidance (Aricescu et al., 2002; Johnson et al., 2006). Both PTPσ and LAR, together with PTPδ, belong to the type IIa RPTP (receptor-type protein tyrosine phosphatase) family (Tonks, 2006). They are type I transmembrane proteins that each possess three immunoglobulin-like domains and typically four or eight fibronectin repeats. Two tandem repeats of the PTP domain composed of catalytically active D1 and inactive D2 form the intracellular domain (Tonks, 2006). The catalytic activity is regulated by receptor monomerization and multimerization. As a monomer, the D1 domain is free and active, while the D1 domain is *cis*-inhibited by the D2 domain as a multimer. Both CS and HS share the same binding domain on the first immunoglobulin-like domain of PTPσ and LAR, in which the basic amino acids form clusters at the surface, implying its ionic interaction with the sulfate groups of CS and HS (Aricescu et al., 2002; Shen et al., 2009). The additional binding site at the juxtamembrane domain on PTPσ for HS was also reported (Katagiri et al., 2018). On the basis of biochemical and structural analyses, the “switch model” of PTPσ by HS and CS was proposed, in which HS induced receptor clustering, on the other hand, CS promoted receptor monomerization (Coles et al., 2011). However, how these two sulfated glycans act in such an opposing manner remained unclear, as did the specific substrates for PTPσ that are responsible for forming the dystrophic endbulb.

## Disruption of Autophagy in Dystrophic Endbulb by CS

In our recent work, we tried to explore deeply how CS and HS differentially regulate PTPσ, which ultimately determines axonal fate after injury (Sakamoto et al., 2019). The heterogeneity of glycan sulfation patterns in a single chain of GAG makes it harder to determine which segment binds to PTPσ. To overcome this, we synthesized and prepared a pure CS and HS oligosaccharides library with defined lengths and sulfation patterns. The surface plasmon resonance method was initially used to determine the interactions between CS variants and PTPσ. We found that CS-E, which has a highly proportion of E-unit (GlcA-GalNAc4S,6S), showed the best affinity to PTPσ among the CS variants, CS-A, CS-C, CS-D, and CS-E (Sakamoto et al., 2019). We found that CS-E, which has C4- and C6-sulfation on GalNAc residues, showed the best affinity to PTPσ. We then tested PTPσ with different lengths of CS-E and determined that the minimal binding segment of CS-E was a tetrasaccharide (Figure 1; Sakamoto et al., 2019). It is noteworthy that CS-E is a rare sulfation pattern and accounts for only a small percentage of the total CS in the injuredCNS in mice (Properzi et al., 2005). This means the frequency of CS-E tetrasaccharide can be estimated to be one at most in a single CS chain, and the CS chain is a preferable structure that can induce monomerization and activation of PTPσ (Figure 1). In contrast, with similar methods, we found that HS with one or more sulfate groups was sufficient to interact with PTPσ (Sakamoto et al., 2019). These structures account for nearly half of the total HS in the injured CNS (Properzi et al., 2008). Again, the results imply that the HS chain is preferable for inducing multimerization and inactivation of the receptor. This idea was confirmed by a cell culture experiment and synthetic CS/HS oligosaccharides. Although how these two distinct GAG chains with different levels of sulfation interact with PTPσ is still unclear, these data demonstrated that the frequency of the binding domain, defined by both the sulfation pattern and length in CS and HS, determined the action mode of each glycan on PTPσ.

To understand what a dystrophic endbulb is, and the consequence of PTPσ activation by CS, we deeply observed adystrophic endbulb formed on a CS gradient *in vitro* by electron microscopy. We found that autophagosomes abnormally accumulated in the dystrophic endbulb. Immunostaining with LC3, a specific marker for autophagosome, supported the results. More importantly, autophagosomes also accumulated at the tips of the dissected corticospinal tract in a mouse model of SCI (Sakamoto et al., 2019).

Autophagy is an intracellular degradation system for organelles and cytoplasmic components (Mizushima and Komatsu, 2011). Phagophore, a bowl-like membrane structure which is often formed at the contact site between endoplasmic reticulum and mitochondria, engulfs and encloses these components, producing autophagosome. The autophagosome then fuses with lysosome and is converted to autolysosome. As a consequence, components of autophagosome are delivered into lysosome and degraded by lysosomal enzymes. In axons, autophagy occurs preferentially at the axon tips and autophagosomes are retrogradely transported on microtubules to the cell body, fusing with lysosome during transport (Maday et al., 2012; Maday and Holzbaur, 2014). Because elongating axons have a high turnover of molecules and organelles including cytoskeleton, mitochondria, and membrane, the process seems to be essential to homeostasis for the elongation of axon tips. There are two mechanisms for the accumulation of autophagosomes (1) activation of autophagy and (2) disruption of autophagy flux, especially at the fusion step between autophagosomes and lysosomes. Immunocytochemical analysis using tandem-fluorescent LC3 (Kimura et al., 2007), which can distinguish between autophagosome and autolysosome, revealed that autophagy flux was severely disrupted in the dystrophic endbulb. Importantly, the knockdown of Syntaxin 17, Vamp 8, or Snap 29 by RNA interference, each of which is an essential soluble *N*-ethylmaleimide-sensitive factor attachment protein receptor (SNARE) for autophagosome-lysosome fusion (Itakura et al., 2012), successfully transformed healthy growth cones into dystrophic endbulb-like structures and significantly suppressed axonal elongation in cultured mouse dorsal root ganglion neuron. Consistent with this, treatments of the growth cones with chloroquine or bafilomycin A1, either of which inhibited the fusion between autophagosomes and lysosomes, gave similar results. Taken together, these results clearly demonstrated that disruption of autophagy flux was essential and sufficient for the formation of dystrophic endbulbs (Figure 1).

To link the PTPσ-autophagy axis, we focused on finding the specific substrate for PTPσ that is involved at the fusion step between autophagosomes and lysosomes (Sakamoto et al., 2019). Cortactin is an actin-binding protein thatis required for the process (Lee et al., 2010; Hasegawa et al., 2016). It has several tyrosine phosphorylation sites, among which the tyrosine 421 and 466 phosphorylation sites are essential for its actin-stabilizing activity (Hasegawa et al., 2016). Some of these tyrosine-phosphorylated cortactins are specifically localized to the lysosome surface by the protein-lipid interaction (Hasegawa et al., 2016), where they provide stabilized actin fibers to lysosomes to fuse with autophagosomes. In the primary cultured dorsal root ganglion neurons on a CSPG gradient, reduced phosphorylation at tyrosine 421 of cortactin was observed in the dystrophic endbulb compared to healthy growth cones. Furthermore, silencing cortactin resulted in dystrophic endbulb formation, similar to the case with CS treatment. Thus, we conclude that CS binds to PTPσ and the activated PTPσ dephosphorylates cortactin. This results in the disruption of the completion of the autophagy flux, causing the transformation of the growth cone into a dormant dystrophic endbulb (Figure 1).

## Concluding Remarks

CS and HS, each with different backbones and sulfation patterns, can bind to PTPσ. Currently, it is well known that CS inhibits axonal regeneration while HS promotes it. In a recent study we prepared a library of HS octasaccharides and found that, through these octasaccharides together with CS octasaccharides, PTPσ preferentially interacts with CS-E, a rare sulfation pattern in the natural CS chain, as well as with most HS oligomers bearing sulfate and sulfamate groups. Consequently, short and long stretches of natural CS and HS, respectively, bind to PTPσ. CS activates PTPσ, which dephosphorylates cortactin, a newly identified substrate for PTPσ, and disrupts autophagy flux at the autophagosome-lysosome fusion step. The failure of autophagy flux causes an accumulation of autophagosomes at the growth cone and is sufficient to turn the growth cone into a dystrophic endbulb. As a result, we conclude that the sulfation patterns determine the length of the GAG segment that binds to PTPσ and defines the fate of axonal regeneration through the PTPσ-cortactin-autophagy axis. Our results shed light on the mechanisms by which GAGs function as ligands to cell surface receptors. In addition, the present findings provide a new therapeutic strategy, including glycomimetics. Indeed, we recently showed that enoxaparin, a heparin oligosaccharide medicine clinically used as an anticoagulant, promoted functional recovery in a rat model of SCI, probably through the inactivation of PTPσ (Ito et al., 2021).

We cannot exclude the possibility that other intracellular mediators and mechanisms are involved in the formation of dystrophic endbulbs and in the inhibition of axonal regeneration. For instance, we recently identified a lot of interactors, including cortactin, for PTPσ by the proximity-dependent ligation assay (Gong et al., 2021). Further understanding toward dystrophic endbulbs is still needed to achieve complete regeneration of axons.

## Author Contributions

KS wrote the manuscript. KS, TO, and KK discussed the manuscript. All authors contributed to the article and approved the submitted version.

## Conflict of Interest

The authors declare that the research was conducted in the absence of any commercial or financial relationships that could be construed as a potential conflict of interest.

## References

[B1] Anderson M. A. Burda J. E. Ren Y. Ao Y. O’Shea T. M. Kawaguchi R. (2016). Astrocyte scar formation aids central nervous system axon regeneration. *Nature* 532 195–200. 10.1038/nature17623 27027288 PMC5243141

[B2] Aricescu A. R. McKinnell I. W. Halfter W. Stoker A. W. (2002). Heparan sulfate proteoglycans are ligands for receptor protein tyrosine phosphatase sigma. *Mol. Cell Biol.* 22 1881–1892. 10.1128/mcb.22.6.1881-1892.2002 11865065 PMC135600

[B3] Asher R. A. Morgenstern D. A. Fidler P. S. (2000). Neurocan is upregulated in injured brain and in cytokine-treated astrocytes. *J. Neurosci.* 20 2427–2438. 10.1523/jneurosci.20-07-02427.2000 10729323 PMC6772249

[B4] Benfey M. Aguayo A. J. (1982). Extensive elongation of axons from rat brain into peripheral nerve grafts. *Nature* 296 150–152. 10.1038/296150a0 7063015

[B5] Bradbury E. J. Moon L. D. Popat R. J. King V. R. Bennett G. S. Patel P. N. (2002). Chondroitinase ABC promotes functional recovery after spinal cord injury. *Nature* 416 636–640. 10.1038/416636a 11948352

[B6] Coles C. H. Shen Y. Tenney A. P. Siebold C. Sutton G. C. Lu W. (2011). Proteoglycan-specific molecular switch for RPTPσ clustering and neuronal extension. *Science* 332 484–488. 10.1126/science.1200840 21454754 PMC3154093

[B7] David S. Aguayo A. J. (1981). Axonal elongation into peripheral nervous system “bridges” after central nervous system injury in adult rats. *Science* 214 931–933. 10.1126/science.6171034 6171034

[B8] Dickendesher T. L. Baldwin K. T. Mironova Y. A. Koriyama Y. Raiker S. J. Askew K. L. (2012). NgR1 and NgR3 are receptors for chondroitin sulfate proteoglycans. *Nat. Neurosci.* 15 703–712. 10.1038/nn.3070 22406547 PMC3337880

[B9] Fisher D. Xing B. Dill J. Li H. Hoang H. H. Zhao Z. (2011). Leukocyte common antigen-related phosphatase is a functional receptor for chondroitin sulfate proteoglycan axon growth inhibitors. *J. Neurosci.* 31 14051–14066. 10.1523/JNEUROSCI.1737-11.2011 21976490 PMC3220601

[B10] Frischknecht R. Heine M. Perrais D. Seidenbecher C. I. Choquet D. Gundelfinger E. D. (2009). Brain extracellular matrix affects AMPA receptor lateral mobility and short-term synaptic plasticity. *Nat. Neurosci.* 12 897–904. 10.1038/nn.2338 19483686

[B11] Gogolla N. Caroni P. Lüthi A. Herry C. (2009). Perineuronal nets protect fear memories from erasure. *Science* 325 1258–1261. 10.1126/science.1174146 19729657

[B12] Gong Y. Abudureyimu S. Kadomatsu K. Sakamoto K. (2021). Identification of PTPRσ-interacting proteins by proximity-labelling assay. *J. Biochem.* 169 187–194. 10.1093/jb/mvaa141 33313879

[B13] Hasegawa J. Iwamoto R. Otomo T. Nezu A. Hamasaki M. Yoshimori T. (2016). Autophagosome-lysosome fusion in neurons requires INPP5E, a protein associated with Joubert syndrome. *EMBO J.* 35 1853–1867. 10.15252/embj.201593148 27340123 PMC5007553

[B14] Hughes J. T. (1988). The Edwin Smith Surgical Papyrus: an analysis of the first case reports of spinal cord injuries. *Paraplegia* 26 71–82. 10.1038/sc.1988.15 3045730

[B15] Imagama S. Sakamoto K. Tauchi R. Shinjo R. Ohgomori T. Ito Z. (2011). Keratan sulfate restricts neural plasticity after spinal cord injury. *J. Neurosci.* 31 17091–17102. 10.1523/jneurosci.5120-10.2011 22114278 PMC6623845

[B16] Inatani M. Irie F. Plump A. S. Tessier-Lavigne M. Yamaguchi Y. (2003). Mammalian brain morphogenesis and midline axon guidance require heparan sulfate. *Science* 302 1044–1046. 10.1126/science.1090497 14605369

[B17] Itakura E. Kishi-Itakura C. Mizushima N. (2012). The hairpin-type tail-anchored SNARE syntaxin 17 targets to autophagosomes for fusion with endosomes/lysosomes. *Cell* 151 1256–1269. 10.1016/j.cell.2012.11.001 23217709

[B18] Ito S. Ozaki T. Morozumi M. Imagama S. Kadomatsu K. Sakamoto K. (2021). Enoxaparin promotes functional recovery after spinal cord injury by antagonizing PTPRσ. *Exp. Neurol.* 340 113679. 10.1016/j.expneurol.2021.113679 33662380

[B19] Ito Z. Sakamoto K. Imagama S. Matsuyama Y. Zhang H. Hirano K. (2010). N-acetylglucosamine 6-O-sulfotransferase-1-deficient mice show better functional recovery after spinal cord injury. *J. Neurosci.* 30 5937–5947. 10.1523/JNEUROSCI.2570-09.2010 20427653 PMC6632605

[B20] Johnson K. G. Tenney A. P. Ghose A. Duckworth A. M. Higashi M. E. Parfitt K. (2006). The HSPGs Syndecan and Dallylike bind the receptor phosphatase LAR and exert distinct effects on synaptic development. *Neuron* 49 517–531. 10.1016/j.neuron.2006.01.026 16476662

[B21] Kadomatsu K. Sakamoto K. (2014). Sulfated glycans in network rewiring and plasticity after neuronal injuries. *Neurosci. Res.* 78 50–54. 10.1016/j.neures.2013.10.005 24157431

[B22] Katagiri Y. Morgan A. A. Yu P. Bangayan N. J. Junka R. Geller H. M. (2018). Identification of novel binding sites for heparin in receptor protein-tyrosine phosphatase (RPTPσ): Implications for proteoglycan signaling. *J. Biol. Chem.* 293 11639–11647. 10.1074/jbc.RA118.003081 29880643 PMC6065195

[B23] Kimura S. Noda T. Yoshimori T. (2007). Dissection of the autophagosome maturation process by a novel reporter protein, tandem fluorescent-tagged LC3. *Autophagy* 3 452–460. 10.4161/auto.4451 17534139

[B24] Lee J. Y. Koga H. Kawaguchi Y. Tang W. Wong E. Gao Y. S. (2010). HDAC6 controls autophagosome maturation essential for ubiquitin-selective quality-control autophagy. *EMBO J.* 29 969–980. 10.1038/emboj.2009.405 20075865 PMC2837169

[B25] Maday S. Holzbaur E. L. (2014). Autophagosome biogenesis in primary neurons follows an ordered and spatially regulated pathway. *Dev. Cell* 30 71–85. 10.1016/j.devcel.2014.06.001 25026034 PMC4109719

[B26] Maday S. Wallace K. E. Holzbaur E. L. (2012). Autophagosomes initiate distally and mature during transport toward the cell soma in primary neurons. *J. Cell Biol.* 196 407–417. 10.1083/jcb.201106120 22331844 PMC3283992

[B27] Margolis R. K. Margolis R. U. (1993). Nervous tissue proteoglycans. *Experientia* 49 429–446. 10.1007/bf01923587 8500598

[B28] Mikami T. Yasunaga D. Kitagawa H. (2009). Contactin-1 is a functional receptor for neuroregulatory chondroitin sulfate-E. *J. Biol. Chem.* 284 4494–4499. 10.1074/jbc.M809227200 19075012

[B29] Miyata S. Komatsu Y. Yoshimura Y. Taya C. Kitagawa H. (2012). Persistent cortical plasticity by upregulation of chondroitin 6-sulfation. *Nat. Neurosci.* 15 414–422. 10.1038/nn.3023 22246436

[B30] Mizushima N. Komatsu M. (2011). Autophagy: renovation of cells and tissues. *Cell* 147 728–741. 10.1016/j.cell.2011.10.026 22078875

[B31] Moon L. D. Asher R. A. Rhodes K. E. Fawcett J. W. (2001). Regeneration of CNS axons back to their target following treatment of adult rat brain with chondroitinase ABC. *Nat. Neurosci.* 4 465–466. 10.1038/87415 11319553

[B32] Pizzorusso T. Medini P. Berardi N. Chierzi S. Fawcett J. W. Maffei L. (2002). Reactivation of ocular dominance plasticity in the adult visual cortex. *Science* 298 1248–1251. 10.1126/science.1072699 12424383

[B33] Pizzorusso T. Medini P. Landi S. Baldini S. Berardi N. Maffei L. (2006). Structural and functional recovery from early monocular deprivation in adult rats. *Proc. Natl. Acad. Sci. U S A* 103 8517–8522. 10.1073/pnas.0602657103 16709670 PMC1482523

[B34] Properzi F. Carulli D. Asher R. A. Muir E. Camargo L. M. van Kuppevelt T. H. (2005). Chondroitin 6-sulphate synthesis is up-regulated in injured CNS, induced by injury-related cytokines and enhanced in axon-growth inhibitory glia. *Eur. J. Neurosci.* 21 378–390. 10.1111/j.1460-9568.2005.03876.x 15673437

[B35] Properzi F. Lin R. Kwok J. Naidu M. van Kuppevelt T. H. Ten Dam G. B. (2008). Heparan sulphate proteoglycans in glia and in the normal and injured CNS: expression of sulphotransferases and changes in sulphation. *Eur. J. Neurosci.* 27 593–604. 10.1111/j.1460-9568.2008.06042.x 18279312

[B36] Rauch U. Gao P. Janetzko A. Flaccus A. Hilgenberg L. Tekotte H. (1991). Isolation and characterization of developmentally regulated chondroitin sulfate and chondroitin/keratan sulfate proteoglycans of brain identified with monoclonal antibodies. *J. Biol. Chem.* 266 14785–14801. 10.1016/s0021-9258(18)98755-7 1907283

[B37] Rosenzweig E. S. Salegio E. A. Liang J. J. Weber J. L. Weinholtz C. A. Brock J. H. (2019). Chondroitinase improves anatomical and functional outcomes after primate spinal cord injury. *Nat. Neurosci.* 22 1269–1275. 10.1038/s41593-019-0424-1 31235933 PMC6693679

[B38] Ruschel J. Hellal F. Flynn K. C. Dupraz S. Elliott D. A. Tedeschi A. (2015). Axonal regeneration. Systemic administration of epothilone B promotes axon regeneration after spinal cord injury. *Science* 348 347–352. 10.1126/science.aaa2958 25765066 PMC4445125

[B39] Sakamoto K. Kadomatsu K. (2017). Mechanisms of axon regeneration: The significance of proteoglycans. *Biochim. Biophys. Acta Gen. Subj.* 1861 2435–2441. 10.1016/j.bbagen.2017.06.005 28596106

[B40] Sakamoto K. Ozaki T. Ko Y. C. Tsai C. F. Gong Y. Morozumi M. (2019). Glycan sulfation patterns define autophagy flux at axon tip via PTPRsigma-cortactin axis. *Nat. Chem. Biol.* 15 699–709. 10.1038/s41589-019-0274-x 31061498

[B41] Shen Y. Tenney A. P. Busch S. A. Horn K. P. Cuascut F. X. Liu K. (2009). PTPsigma is a receptor for chondroitin sulfate proteoglycan, an inhibitor of neural regeneration. *Science* 326 592–596. 10.1126/science.1178310 19833921 PMC2811318

[B42] Silver J. Miller J. H. (2004). Regeneration beyond the glial scar. *Nat. Rev. Neurosci.* 5 146–156. 10.1038/nrn1326 14735117

[B43] Smith-Thomas L. C. Fok-Seang J. Stevens J. Du J. S. Muir E. Faissner A. (1994). An inhibitor of neurite outgrowth produced by astrocytes. *J. Cell Sci.* 107(Pt 6), 1687–1695. 10.1242/jcs.107.6.1687 7962209

[B44] Snow D. M. Lemmon V. Carrino D. A. Caplan A. I. Silver J. (1990). Sulfated proteoglycans in astroglial barriers inhibit neurite outgrowth in vitro. *Exp. Neurol.* 109 111–130. 10.1016/s0014-4886(05)80013-5 2141574

[B45] Snow D. M. Watanabe M. Letourneau P. C. Silver J. (1991). A chondroitin sulfate proteoglycan may influence the direction of retinal ganglion cell outgrowth. *Development* 113 1473–1485. 10.1242/dev.113.4.1473 1811954

[B46] Tom V. J. Steinmetz M. P. Miller J. H. Doller C. M. Silver J. (2004). Studies on the development and behavior of the dystrophic growth cone, the hallmark of regeneration failure, in an in vitro model of the glial scar and after spinal cord injury. *J. Neurosci.* 24 6531–6539. 10.1523/JNEUROSCI.0994-04.2004 15269264 PMC6729861

[B47] Tonks N. K. (2006). Protein tyrosine phosphatases: from genes, to function, to disease. *Nat. Rev. Mol. Cell Biol.* 7 833–846. 10.1038/nrm2039 17057753

[B48] Tran A. P. Silver J. (2021). Cathepsins in neuronal plasticity. *Neural. Regen Res.* 16 26–35. 10.4103/1673-5374.286948 32788444 PMC7818855

[B49] Tran A. P. Sundar S. Yu M. Lang B. T. Silver J. (2018). Modulation of Receptor Protein Tyrosine Phosphatase Sigma Increases Chondroitin Sulfate Proteoglycan Degradation through Cathepsin B Secretion to Enhance Axon Outgrowth. *J. Neurosci.* 38 5399–5414. 10.1523/JNEUROSCI.3214-17.2018 29760175 PMC5990985

[B50] Wang L. Denburg J. L. (1992). A role for proteoglycans in the guidance of a subset of pioneer axons in cultured embryos of the cockroach. *Neuron* 8 701–714. 10.1016/0896-6273(92)90091-q 1567620

[B51] Warren P. M. Steiger S. C. Dick T. E. MacFarlane P. M. Alilain W. J. Silver J. (2018). Rapid and robust restoration of breathing long after spinal cord injury. *Nat. Commun.* 9:4843. 10.1038/s41467-018-06937-0 30482901 PMC6258702

